# Nanoscopic Characterization of Cell Migration under Flow Using Optical and Electron Microscopy

**DOI:** 10.1021/acs.analchem.2c04222

**Published:** 2023-01-10

**Authors:** Abdullah Alghamdi, Amar Tamra, Aigerim Rakhmatulina, Shuho Nozue, Asma S. Al-Amoodi, Mansour M. Aldehaiman, Ioannis Isaioglou, Jasmeen S. Merzaban, Satoshi Habuchi

**Affiliations:** Biological and Environmental Science and Engineering Division, King Abdullah University of Science and Technology, Thuwal 23955-6900, Saudi Arabia

## Abstract

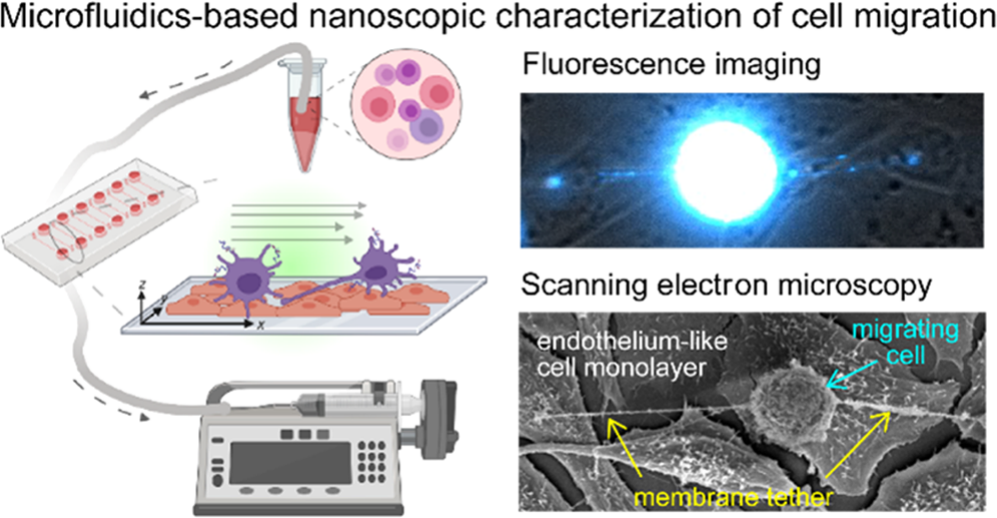

Hematopoietic stem/progenitor cell (HSPC) and leukemic cell homing is an important biological phenomenon that takes place through essential interactions with adhesion molecules on an endothelial cell layer. The homing process of HSPCs begins with the tethering and rolling of the cells on the endothelial layer, which is achieved by the interaction between selectins on the endothelium to the ligands on HSPC/leukemic cells under shear stress of the blood flow. Although many studies have been based on in vitro conditions of the cells rolling over recombinant proteins, significant challenges remain when imaging HSPC/leukemic cells on the endothelium, a necessity when considering characterizing cell-to-cell interaction and rolling dynamics during cell migration. Here, we report a new methodology that enables imaging of stem-cell-intrinsic spatiotemporal details during its migration on an endothelium-like cell monolayer. We developed optimized protocols that preserve transiently appearing structures on HSPCs/leukemic cells during its rolling under shear stress for fluorescence and scanning electron microscopy characterization. Our new experimental platform is closer to in vivo conditions and will contribute to indepth understanding of stem-cell behavior during its migration and cell-to-cell interaction during the process of homing.

## Introduction

Hematopoiesis is the process of blood cellular component formation. It occurs during embryonic development and continues throughout adulthood to produce and replenish the blood system.^1−3^ Hematopoietic stem-cell delivery to specific sites in the body is central to many physiological functions from immunity to cancer metastasis.^2,3^ During the process of “homing”, HSPCs extravasate through the endothelial cells from peripheral blood to the bone marrow and start repopulating it by producing hematopoietic lineage cells including red blood cells, white blood cells, platelets, granulocytes, and erythrocytes.^4−8^ The process of homing is initiated by tethering and rolling of the cells in flow followed by firm adhesion and transmigration into the tissue.^9,10^ This is achieved by the cell’s interactions with the surface of the endothelium under the presence of external shear forces.^11−13^ Cell surface glycoproteins such as CD44 and PSGL-1 interact with receptors expressed on endothelial cells, E- and P-selectins, and integrins.^7,14−21^ This selectin–ligand interaction promotes the transient formation of membrane tethers and slings by HSPCs, resulting in their rolling along the endothelium at a shear stress of several dynes cm^–2^ generated by the blood flow.^2,3,22^

To date, extensive studies have been conducted to understand the homing mechanisms of HSPCs, including initial rolling and tethering steps.^6,23,24^ The most common and widespread technique to image and characterize rolling HSPCs has been a fluidics-based in vitro cell-rolling assay which mimics the cell-rolling behavior by flowing HSPCs in a fluidic chamber whose surface is coated with adhesion molecules.^2,13,18−21,25−30^ Integration of advanced fluorescence imaging techniques to the microfluidics-based cell-rolling assay has enabled visualization and characterization of nanoscopic spatiotemporal dynamics of adhesion molecules as well as cell surface architecture during cell rolling.^2,3^ Using this platform, we have demonstrated that the initial step of homing is regulated by spatial localization of the selectin ligands to membrane tethers and slings and their fast motion along these structures is due to the absence of anchoring to the underlying actin cytoskeleton.^2,3^ Alternatively, the migration behavior of HSPCs has been investigated at the single-cell level by engineering endothelial vascular networks that closely mimic the bone marrow microenvironment.^31^ Cell rolling^32^ as well as cell extravasation of leukocytes^32−35^ and HSPCs^4,36−39^ across the endothelium have been studied using this approach. In vivo imaging has also been used for capturing the cell migration in bone marrow.^40^

HSPCs rolling on an endothelial cell layer have enabled single-cell-level imaging and characterization of homing under conditions that closely mimic the bone marrow microenvironment. However, nanoscopic subcellular-level characterization (e.g., membrane tethers and sling formation, shear force-dependent selectin–ligand interactions, and spatiotemporal dynamics of selectin ligands) using endothelial cell layers, particularly under flow conditions, remain challenging although they play key roles in HSPC homing.

**Figure 1:**
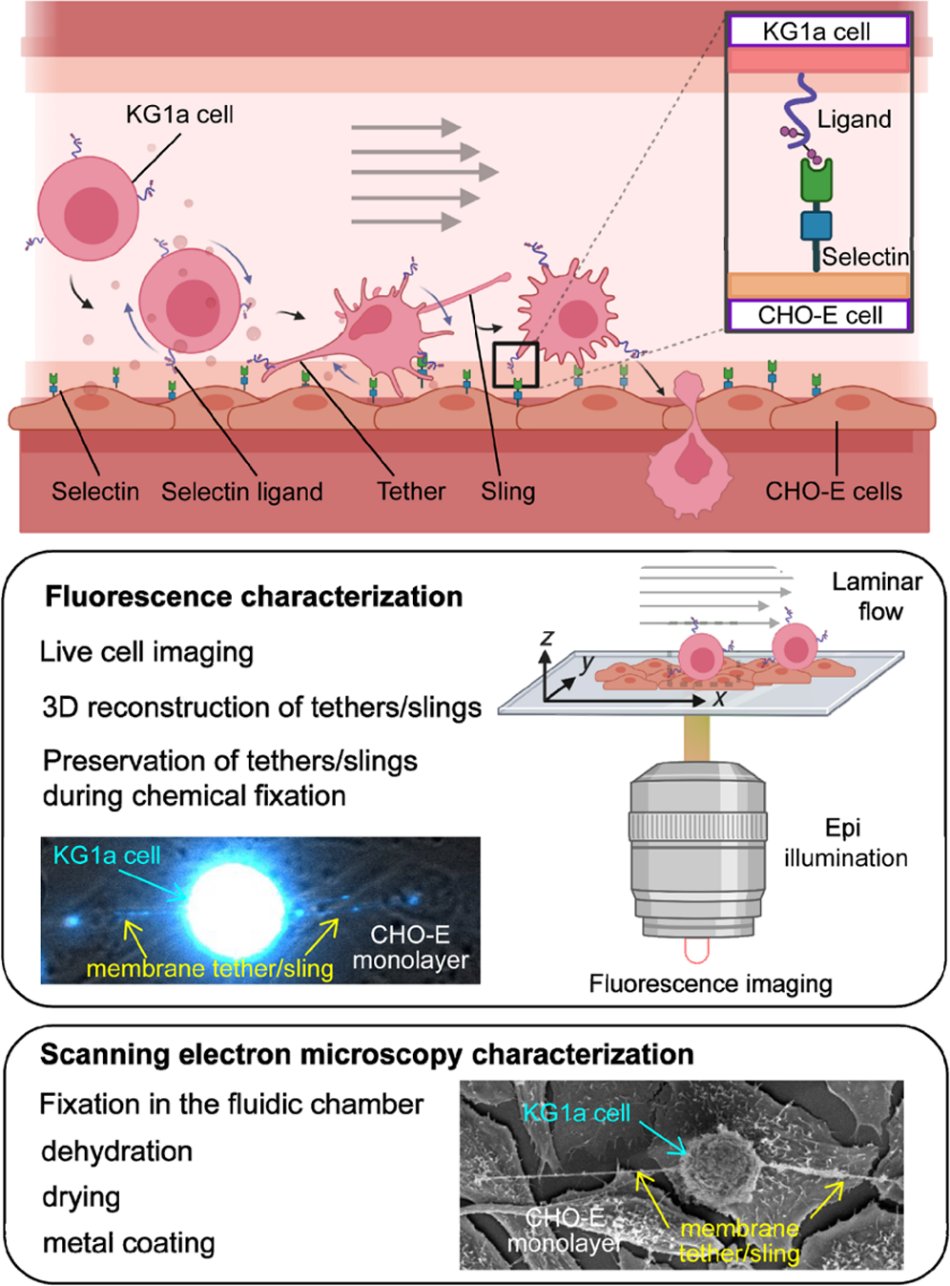
Schematic illustration describing the experimental configuration.

In this study, we developed microfluidics-based methodologies for imaging and characterization of spatiotemporal HSPC migration over a monolayer of Chinese hamster ovary (CHO) cells that express E-selectin on their surface,^41^ which is a condition very close to in vivo HSPC rolling (Figure 1). Live-cell fluorescence wide-field microscopy and confocal microscopy on the rolling cells revealed the formation of membrane tethers and slings and spatial localization of CD44 on the rolling cells. We also showed that our experimental protocol enables the preservation of transiently existing fragile structures (e.g., membrane tethers and slings) formed only under flow conditions throughout the fixation process of rolling cells. This allowed us to apply scanning electron microscopy (SEM) as a new tool for capturing and nanoscopic characterization of these transient structures (Figure 1).

## Experimental Section

### Cells and Treatments

Chinese Hamster Ovary cell lines transfected or not to express human E-selectin (CHO-E and CHO-K, cells transfected with mock plasmid, cells)^41^ were maintained in Dulbecco’s modified Eagle’s medium (DMEM; Gibco), supplemented with 10% fetal bovine serum, penicillin (100 U mL^–1^)/streptomycin (100 μg mL^–1^), 1% sodium pyruvate, and 10% nonessential amino acids. KG1a cells, a human acute myelogenous leukemia cell line (ATCC), were maintained in Iscove’s modified Dulbecco’s medium (IMDM; Gibco) supplemented with 10% fetal bovine serum, penicillin (100 U mL^–1^), and streptomycin (100 μg mL^–1^). Both cell lines were maintained at 37 °C in a humidified atmosphere containing 5% CO_2_. The viability of the cells was routinely checked by trypan blue staining or by calcein AM dye. Native fibronectin purified from human plasma was purchased from Sigma. An E-selectin blocking antibody anti-E-Selectin/CD62E (BBIG) was purchased from Utech. An E-selectin blocking antibody anti-E-selectin (H18-7) was purified from hybridoma mouse H18/7 (ATCC: HB-11684).

### Cell Culture in a Microfluidic Channel

We used microfluidic chambers (channel width, 3.8 mm; channel length, 17 mm; channel height, 0.4 mm; μ-slide VI 0.4) purchased from Ibidi (Martinsried, Germany). We chose either polymer culture-treated coverslips or glass coverslips, depending on the type of subsequent microscopy used. Regardless of the used coverslip’s material, the channels were coated with Fibronectin (75 μg mL^–1^) to enhance the attachment of CHO cells. After preparing the cell suspension at a density of 2 × 10^6^ cells mL^–1^, we injected it into the microchannel from the inlet and cultured it at 37 °C to promote cell adhesion. After at least 1 h, we added culture media to the inlet and outlet of the microchannel, preventing evaporation and change in salt concentrations affecting cell viability. Cells were then kept in culture for at least 24 h before using them for subsequent experiments. Cell viability in the chambers was routinely verified with calcein AM staining (Sigma-Aldrich).

### Cell-Rolling Assay

The inlet of the fluidic chamber was connected to the cell-rolling buffer (Hanks’ Balanced Salt Solution (HBSS) containing 1% bovine serum albumin (Sigma)) and to the KG1a cell suspension. The outlet of the chamber was connected to a programmable syringe pump (PHD ULTRA, Harvard Apparatus). Silicone tubings (0.8 mm), male Luer connectors, and female Luer Lock connectors, used to set up the microfluidic pathways, were all purchased from Ibidi GmbH. Before injecting KG1a cells, we flowed the cell-rolling buffer into the chamber to wash the CHO cells and to allow equilibration of the flow path. The cell-rolling assay was conducted using a protocol similar to that used in our previous studies.^2,3^ Briefly, KG1a cells were perfused in the microfluidic pathway and left to rest for 60 s to allow them to settle and interact with the CHO-E monolayer. The flow of the cell-rolling buffer was then resumed with fluid shear stress (FSS) values of 0.25, 0.5, 1, 2, and 4 dyne cm^–2^ (0.025, 0.05, 0.1, 0.2, and 0.4 Pa). We mounted the microfluidic chamber on an inverted bright-field optical microscope (CXK41, Olympus) equipped with a 20× objective lens (LCAch N 20×, Olympus). A video of the KG1a cells rolling over the CHO cell monolayer was recorded using a CCD camera (XC10, Olympus) and CellSens software (Olympus). Shear stress-dependent cell-rolling behavior was analyzed by TrackMate Fiji, an ImageJ plugin. We used the LAP (Linear Assignment Problem) tracker to localize and track cells. Then, we extracted two parameters, displacement of the trajectory and the cell-rolling velocity.

### Surface Deposition of Recombinant E-Selectin

A commercially available microfluidic chamber, μ-slide VI 0.5 glass bottom (3.8 mm width and 0.54 mm height, ibidi GmbH) was coated by a recombinant homodimeric human (rh) E-selectin (SELE, human protein, recombinant hlgG-Fc, His TAG, Sino Biological) to conduct the cell-rolling assay and fluorescence imaging experiments of KG1a cells on the recombinant E-selectin. The surface of the chambers was coated by a sequential deposition of protein-A and rh E-selectin using a protocol that we reported previously.^3^ The chambers were washed at least three times using 120 μL of HBSS before perfusing ether fluorescently labeled or nonlabeled KG1a cells. The surface density of the deposited rh E-selectin was estimated by comparing the fluorescence intensity obtained from the immunolabeled rh E-selectins in a unit area (1 μm^2^) of the surface and that obtained by single immunolabeled rh E-selectin molecules (Figure S4).^3^ The rh E-selectins were immunolabeled by monoclonal rabbit anti-human E-selectin antibody followed by Alexa-Fluor (AF) 647 dye-conjugated anti-rabbit antibody; polyclonal.

### E-Selectin Surface Density on CHO-E Cells

To measure the surface density of E-selectin expressed on CHO-E cells, we immunolabeled E-selectins and captured fluorescence images.

The number of E-selectin molecules was quantitatively estimated by measuring the fluorescence intensity per unit area (1 μm^2^) per cell. To achieve this, first, live CHO-E cells cultured in a glass-bottom microfluidic chamber were fixed with 100 μL of 4% paraformaldehyde and then blocked with 100 μL of 1% casein in PBS (ThermoFisher) at room temperature for 40 min. Next, the cells were immunolabeled with 100 μL of 10 μg mL^–1^ monoclonal primary rabbit anti-human E-selectin (Anti CD62E clone 208) in 1 × HBSS and 1% BSA for 40 min on ice. Then, 100 μL of 5 μg mL^–1^ polyclonal secondary anti-rabbit antibody conjugated to AF-647 in 1 × HBSS and 1% BSA were used for immunolabeling the primary antibodies. After that, a three-dimensional (3D) (1 μm *z*-step size) fluorescence image of the stained CHO-E cells was captured in real time as KG1a cells rolling on the top of the endothelial layer using either wide-field custom-built setup (see below) or commercially available confocal microscopy (Lieca SP8). Finally, the obtained multiple planes 3D of single cells were stacked at maximum intensity projection and analyzed using ImageJ. The autofluorescence intensity was subtracted from the measured integrated intensity per pixel, then converted into molecules by dividing it by the integrated intensity obtained from a single immunolabeled rh E-selectin. The same experimental method could be performed on fixed cells rather than live cells.

To estimate the intensity profile of a single rh E-selectin molecule, we deposited 100 μL of (0.001 μg mL^–1^) rh E-selectin on an uncoated surface. Then, the surface-deposited rh E-selectin was incubated with the monoclonal rabbit anti-human E-selectin antibody followed by Alexa-Fluor (AF) 647 dye-conjugated anti-rabbit antibody. Image acquisition parameters were kept consistent throughout the surface E-selectin density measurements.

### Fluorescence Labeling of KG1a Cells

Immunostaining of the KG1a cells for fluorescence imaging was conducted using the protocols that we reported previously with some modifications.^3^ For the live-cell real-time imaging, the KG1a cells were first blocked with a Fc receptor blocker reagent for 1 h in an ice bath. The sample was then incubated with the primary antibody (mouse anti-human CD44 antibody in 2% BSA, 5 mg mL^–1^) followed by the secondary antibody (AF-647-conjugated goat anti-mouse secondary antibody in 2% BSA, 5 mg mL^–1^). The immunostained sample was flowed into the fluidic chamber in a way similar to that for the cell-rolling assay. We used a cold FluoroBrite DMEM including 1 mM CaCl_2_ and 1% BSA as a perfusion buffer. To capture immunofluorescence images of fixed KG1a cells after rolling on the CHO-E cell monolayer, a fixation solution of 4% paraformaldehyde and 0.2% glutaraldehyde was auto-perfused for a few minutes to arrest and fix the cells inside the chamber.

### Fluorescence Microscopy

Wide-field fluorescence imaging experiments were conducted using a home-built fluorescence microscopy setup that we reported previously.^42−44^ Briefly, a continuous-wave (CW) solid-state laser operating at 638 nm (60 mW, Cobolt, MLD) was introduced to the inverted microscope (Olympus, IX71) from its backside port through a focusing lens (*f* = 300 mm; Thorlabs). The samples were illuminated by an epifluorescence configuration through different types of objective lenses (Olympus, 60 × NA = 1.3, UPLSAPO60XS2, silicone oil immersion, or 40 × NA = 1.25, UPLSAPO40XS, silicon oil immersion). The fluorescence emitted from the samples was captured by the same objective, separated from the illumination light by the dichroic mirror, passed an emission bandpass filter, and detected by an EMCCD camera (Andor Technology, iXon3 897). The image acquisition was utilized using the Andor iQ3 software. 3D fluorescence images were obtained by recording epifluorescence images of the cells at different *Z*-axis positions (0.5–1.0 μm step size) using a piezo objective scanner (PI PIFPC P721).^3^ 3D images were reconstructed using the ImageJ plugin. Confocal fluorescence microscopy imaging experiments on the immunolabeled KG1a cells were performed using an inverted confocal microscope (Leica SP8X WLL). Fluorescence images were captured using the ZEN software platform (LAS X Life Science) with a glycerol immersion objective lens (63×, 1.4NA, HC PL APO CS2) and HyD detectors.

### Sample Preparation for Scanning Electron Microscopy

CHO-E cells were cultured on 75 μg mL^–1^ fibronectin-coated glass slides at a concentration of 2 × 10^6^ mL^–1^ cells per glass slide. The glass slides were handled in six-well plates for the convenience of washing and handling steps. Once confluent, the cells were fixed with 2–2.5% glutaraldehyde in 0.1 M cacodylate buffer (pH 7.2–7.4) for overnight incubation at 4 °C. In the case of experiments of KG1a rolling over the CHO-E cell monolayer, cells were fixed after the rolling was complete at the shear stresses of either 1 or 2 dyne cm^–2^, i.e., glutaraldehyde solution was added immediately after the rolling buffer. The sample was sealed by parafilm to avoid any sample evaporation. Following three rinses with 0.1 M cacodylate buffer, the cells were post-fixed in 1% osmium tetroxide/0.1 M cacodylate buffer for 1 h at room temperature. The coverslips were then thoroughly washed with dH_2_O and then dehydrated with an ethanol gradient (30, 50, 70, 90, 100, 100%) before being transferred to a critical point dryer apparatus (CPD 300, Leica) and dried using carbon dioxide as the transitional solvent. Coverslips were then mounted on aluminum stubs with a double-sided carbon adhesive and coated with 4 nm of platinum (K575X Sputter Coater, Quorum). Images were taken using SEM (Thermo Fischer Teneo VS) at 5 kV. Samples with KG1a cells resting on a glass slide were prepared by a previously published protocol.^3^ Analysis of KG1a cell structures, tethers, and slings was done on ImageJ.

### Statistics

Statistical significance was assessed by student’s *t*-test (assuming two-tailed distribution and two-sample unequal variance). All experiments were repeated at least three times to ensure reproducibility. All of the single-cell fluorescence microscopy images reported in this study are representative examples of multiple (*n* > 3) independent experiments.

## Results and Discussion

In this study, we aimed to develop methodologies to visualize and characterize the rolling behavior of KG1a cells on a monolayer of CHO-E cells using fluorescence microscopy and scanning electron microscopy (SEM). This includes proper expression of E-selectin on CHO-E cells, culturing a monolayer of CHO-E cells in the fluidic chamber that is resistant to shear stress, fluorescence imaging of KG1a cells through the layer of CHO-E cells, preservation of cellular architectures during the fixation, and transfer of the fixed cells to SEM (Figure 1).

### Culturing CHO-E Cells in a Fluidic Chamber

**Figure 2:**
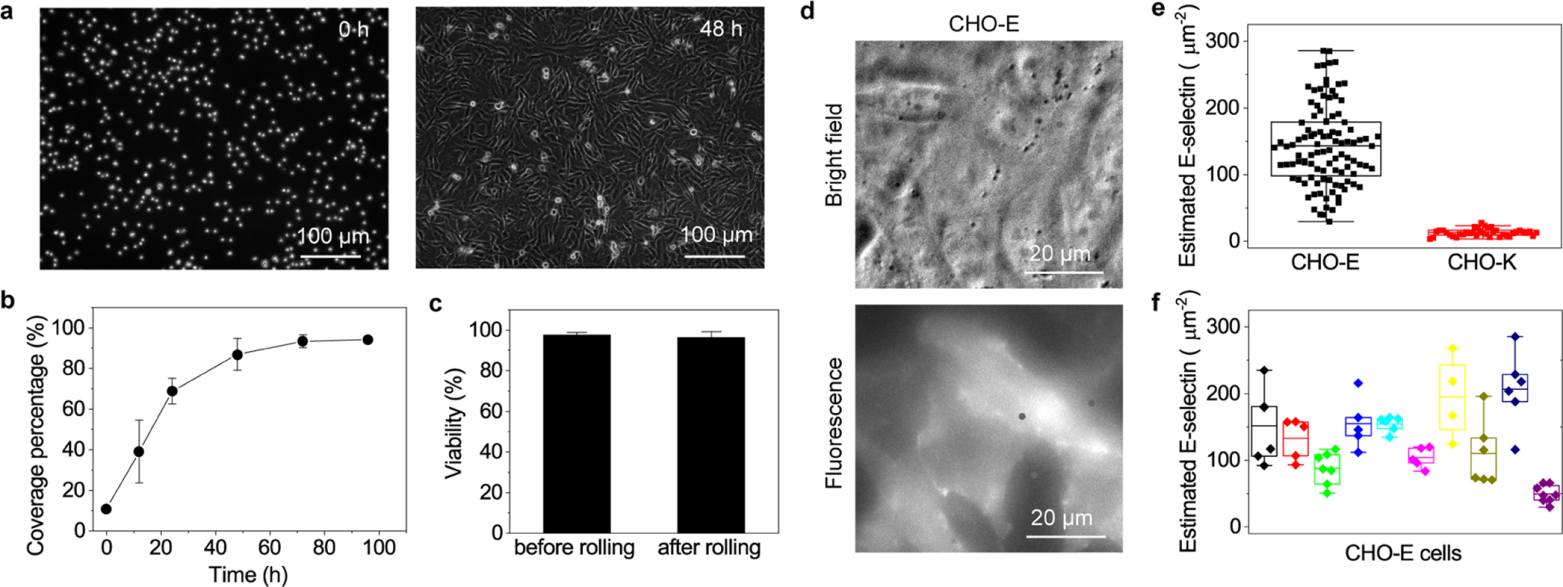
Characterization of the endothelium-like CHO-E monolayer. (a) Bright-field images of cultured CHO-E cells in the fluidic chamber. The cells were seeded over a glass-bottom fibronectin-coated surface. (b) Time-lapse experiment of CHO-E cell culture confluence. Total surface coverage is shown in percentage. (c) Viability of CHO-E cells before and after applying 2 dyne cm^–2^ shear stress for 10 min. (d) Bright-field and fluorescence images of fixed and stained CHO-E cells. E-selectin surface receptors were immunolabeled using primary antibodies and secondary AF-647 conjugated antibodies. (e) Box plots showing the number of E-selectin expressed on the surface of CHO-E and CHO-K cells. Each spot represents mean number of E-selectin in each cell. (f) Box plots showing the number of E-selectin at different locations on each CHO-E cell. Each spot represents the number of E-selectin in a different location on the same CHO-E cell. Data on total 10 CHO-E cells are displayed. Error bars represent ± standard deviation (SD) determined by *n* = 3 independent experiments in panels (b) and (c).

CHO-E cells should form a densely packed monolayer within the fluidic chamber to achieve optimal cell rolling. Figure 2a shows surface coverage by the CHO-E cells after the cells were injected into the fibronectin-coated chamber. The coverage gradually increased and reached 94% after culturing for 48 h (Figures 2a and S1). At this time point, most of the CHO-E cells (at least 92% of the cells) adhered to the surface of the chamber and formed a monolayer (Figure 2b). The viability of the CHO-E cells in the fluidic chamber at this time point was ∼98% (Figures 2c and S2), demonstrating high viability of the cells in the chamber. We conducted subsequent experiments only when the surface coverage of the CHO-E cells exceeded 92%.

During the cell-rolling experiment, shear stresses are exerted not only to rolling cells (i.e., KG1a cells) but also to the CHO-E cells. We did not observe the detachment of any fraction of the CHO-E cells after flowing the cell-rolling buffer for 10 min at the shear stress of 2 dyne cm^–2^ (Figure S3). This confirmed that the surface coverage by the CHO-E cells was maintained throughout the rolling experiment.

The surface density of E-selectin is one of the important factors that affect the cell-rolling behavior. We estimated the E-selectin density on each CHO-E cell in the monolayer (Figure 2d,e). A negative control (i.e., CHO cells that do not express human E-selectin, CHO-K) showed negligible E-selectin on the cell surface (Figures 2e and S4). In contrast, the estimated E-selectin on the CHO-E surface was, on average, 140 molecules per μm^–2^, with relatively large cell-to-cell differences (Figure 2d,e). We also observed inhomogeneous distribution of E-selectin within single cells (Figure 2f). Nevertheless, the mean density of E-selectin on the surface of the CHO-E cells in the monolayer (140 molecules per μm^2^) is similar to that on endothelial cells in vivo after stimulation,^45^ suggesting that rolling behaviors of KG1a cells can be characterized under physiologically relevant conditions.

### Rolling Behavior of KG1a Cells on a CHO-E Monolayer

**Figure 3:**
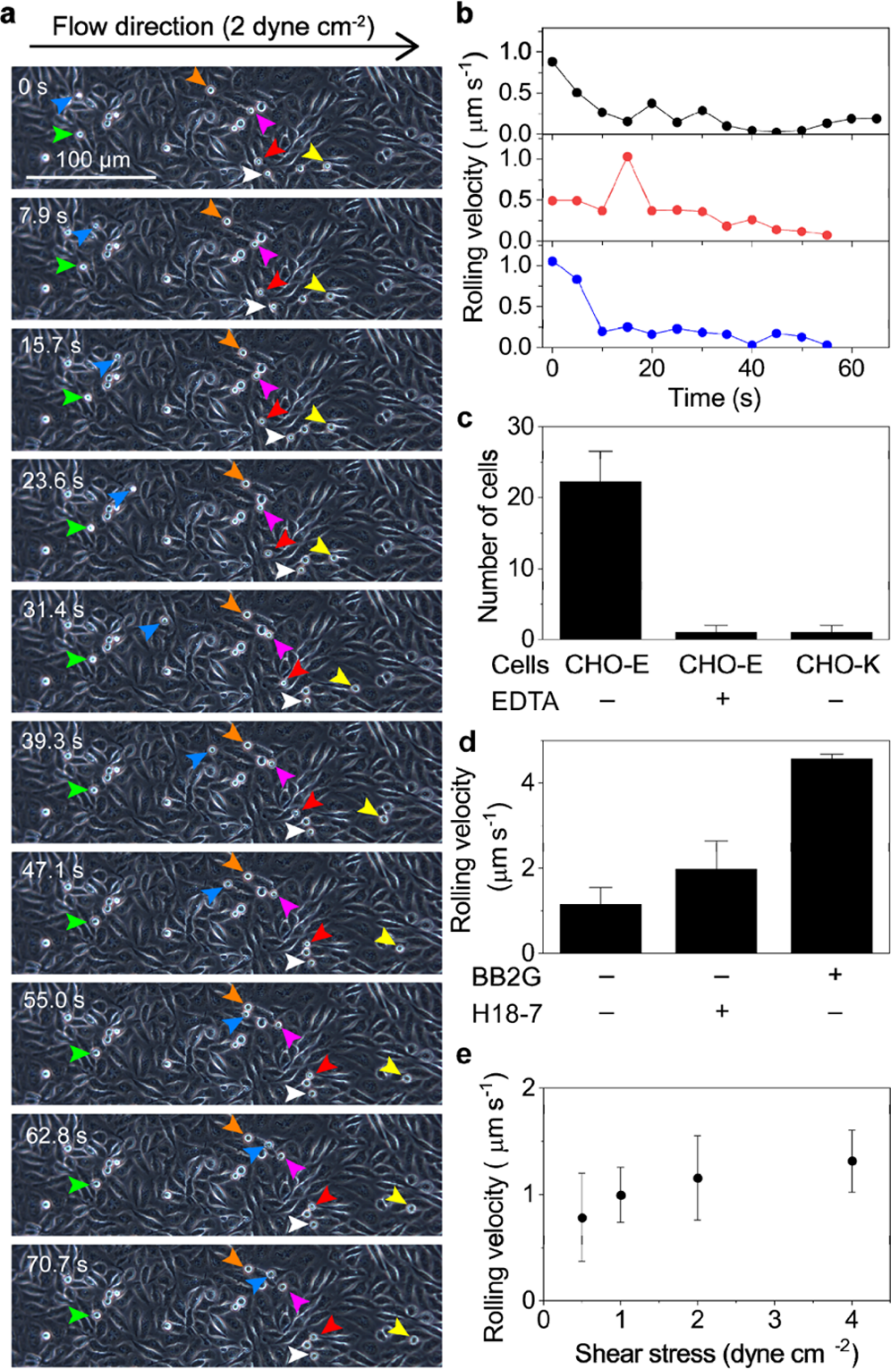
Rolling behavior of KG1a cells on CHO-E monolayer. (a) Time-lapse bright-field images of single KG1a cells rolling over a CHO-E monolayer at a shear stress of 2 dyne cm^–2^. (b) Frame-to-frame single-cell velocity over 70 s time period. (c) Number of adhered then rolled KG1a cells over CHO-E and CHO-K monolayers in the presence or absence of EDTA. (d) Effect of the blocking of active binding sites on E-selectin on the time-averaged rolling velocity of KG1a cells on the CHO-E monolayer. The active binding sites were blocked by two different blocking antibodies, BB2G and H18-7. (e) Time-averaged rolling velocity of KG1a cells over CHO-E monolayer and rh E-selectin deposited on a glass surface with different wall shear stress values. Error bars represent ±SD determined by *n* = 3 independent experiments in panels (c)–(e).

We used KG1a cells, a human leukemic progenitor cell line, as a working model of HSPCs.^41,46^ Rolling behaviors of the KG1a cells were captured by perfusing the KG1a cells in the microfluidic chambers whose surface was covered by the monolayer of CHO-E cells at physiologically relevant shear stresses (0.25–4 dyne cm^–2^). Time-lapse images clearly show that a fraction of the perfused KG1a cells displayed rolling behavior on the monolayer of CHO-E cells (Figure 3a,b). The number of the KG1a cells rolled on the monolayer of CHO-E cells decreased significantly when 10 mM ethylenediamine tetraacetic acid (EDTA) was added to the cell-rolling buffer that inhibits E-selectin–ligand binding (Figure 3c and Videos S1–S3).^3^ Similarly, the monolayer of CHO-K cells (that do not express E-selectin) did not support the tethering and rolling of the KG1a cells (Figure 3c). Further, we observed a 2- to 4-fold increase in the rolling velocity of the KG1a cells upon treating the cells with E-selectin-blocking antibodies (Figure 3d). These results demonstrated that the rolling of the KG1a cells on the CHO-E cells is mediated by specific binding between E-selectin and its ligands.

The rolling velocity of the KG1a cells on the CHO-E cells increased with the applied shear stress (Figure 3e). However, we observed only a slight dependence of the shear stress, which is in contrast to the linear dependence of the rolling velocity to the shear stress observed in a previous study on cell rolling over rh E selection.^2^ The difference in the cell-rolling behavior could be explained by the existence of the cellular architectures (e.g., elastic properties of the endothelium-like CHO-E cell monolayer).^47−49^

### Fluorescence Imaging and Characterization of KG1a Rolling over a CHO-E Monolayer

Capturing the dynamics of tethers and slings that occur in three-dimensional (3D) space in the time scale of milliseconds is experimentally challenging.^27,50^ We previously reported that 3D fluorescence images of tethers and slings formed on KG1a cells rolling over rh E-selectin can be captured by reconstructing 3D images by recording epifluorescence images at different *Z*-axis positions.^3^ While this method does not capture the 3D images of the cell body due to the out-of-focus fluorescence, the method can capture the 3D images of tethers and slings with spatial resolution limited by the diffraction of light. In this study, we applied a similar method to capture the dynamic behavior of tethers and slings on KG1a cells rolling over the CHO-E monolayer. To avoid refractive index mismatching, we used a silicone immersion objective lens for this experiment.

**Figure 4:**
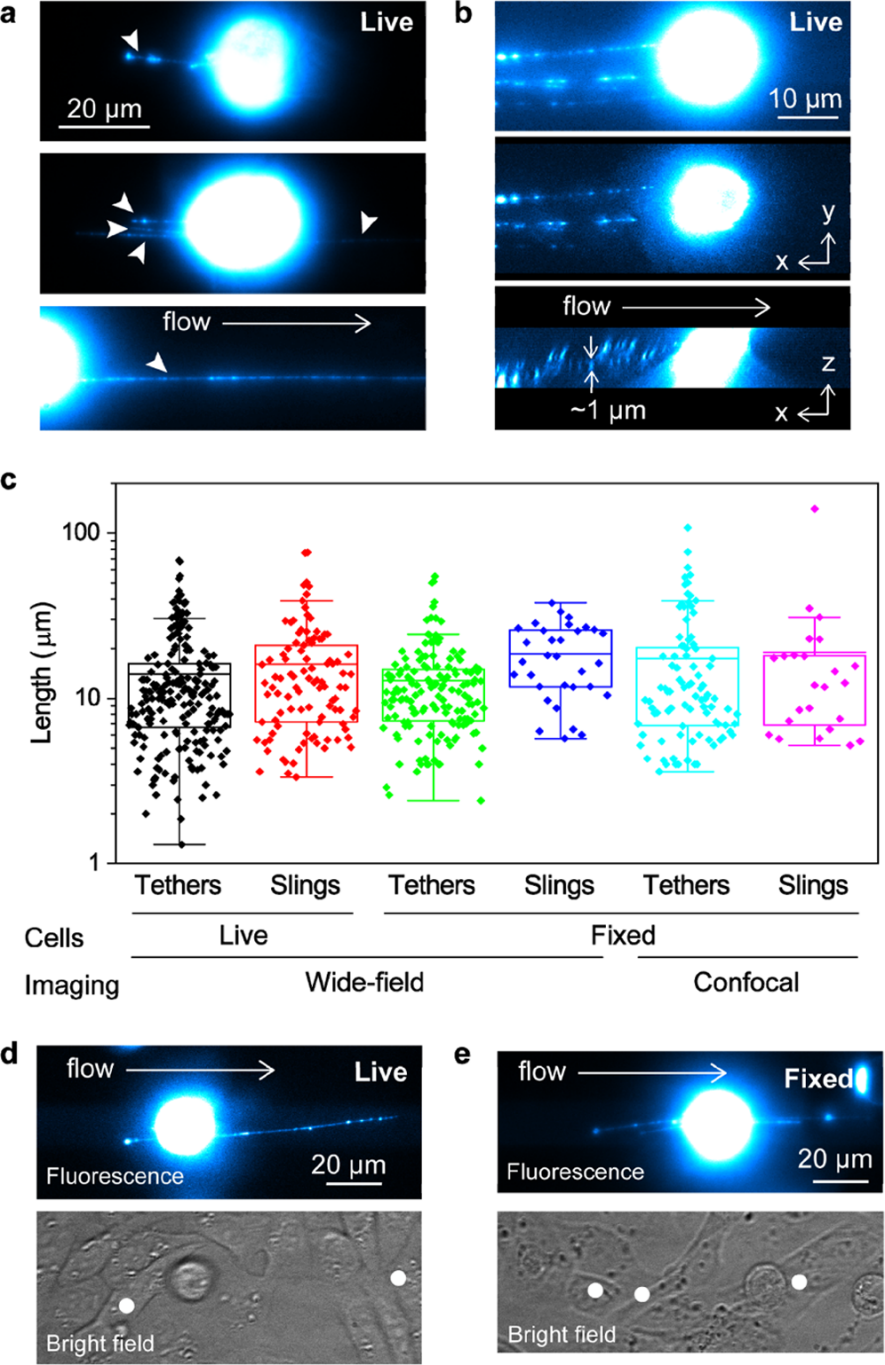
Fluorescence characterization of membrane tethers and slings transiently formed on KG1a cells during the cell rolling over CHO-E monolayer. (a) Fluorescence images of live KG1a cells (immunolabeled against CD44 by Alexa-Fluor (AF)-647 dye-conjugated antibody) captured during cell rolling on the CHO-E monolayer. The arrowheads show tethers and slings. (b) 3D view of the tethers formed on a live KG1a cell rolling on the CHO-E monolayer by CD44. Side view (bottom) and top view (middle) of the 3D reconstructed fluorescence image of CD44 (immunolabeled by AF-647 dye-conjugated antibody) captured during cell rolling on the CHO-E monolayer. The top panel shows a 2D projection of the 3D image. (c) Box plots showing the length of individual tethers and slings on live and fixed KG1a cells rolled on the CHO-E monolayer. The tethers and slings were captured by wide-field or confocal fluorescence microscopy. Fluorescence (top, immunolabeled by AF-647 dye-conjugated antibody) and bright-field (bottom) images of a (d) live KG1a cell rolling on the CHO-E monolayer and (e) KG1a cell fixed after rolling on the CHO-E monolayer. The white dots in the bright-field images show the tethering points. All of the images of the rolling cells were captured at a shear stress of 2 dyne cm^–2^.

A selectin ligand expressed on the KG1a cells, CD44, was immunolabeled by Alexa-Fluor (AF)-647 dye-conjugated antibody to fluorescently visualize the transiently appearing membrane structures (i.e., tethers and slings).^3^ Fluorescence images of the KG1a cells rolled over the CHO-E monolayer clearly showed the formation of membrane tethers and slings (Figure 4a). The fluorescence images also showed that CD44 distributes contiguously along the entire tethers and slings. These findings are consistent with the previous observation on KG1a cells rolled over the rh E-selectin surface.^3^ 3D reconstructed images of the KG1a cells show that the width of the tethers along the axial axis (∼1.0 μm, Figure 4b) is slightly larger than the theoretically calculated depth of the field of the experimental setup (∼0.54 μm),^51^ demonstrating that the 3D images are captured with a minimum effect of the underlying CHO-E monolayer. The average lengths of the tethers and slings were 14 and 16 μm, respectively (Figure 4c). These values are close to the length of the tethers and slings formed during the KG1a cell rolling over rh E-selectin (17 and 16 μm for tethers and slings, respectively).^3^ Tethers and slings longer than 100 μm were occasionally observed (Figures 4c and S5). Importantly, the superimposed bright-field and fluorescence image of KG1a cells rolling over the CHO-E monolayer clearly revealed that the membrane tethers are anchored to CHO-E cells (Figure 4d). Together with the results of the cell-rolling experiments, these data unambiguously demonstrate that the tethers are formed through the binding of selectin ligands to E-selectin on the CHO-E cells. The result also strongly indicates that the formation of the tethers and slings during the tethering and rolling of the KG1a cell is relevant to in vivo cell rolling occurring during the initial step of homing.

While optical microscopy techniques have revealed the importance of membrane tethers and slings and associated spatiotemporal dynamics of selectin ligands on these structures for cell rolling,^3,27,52,53^ they could not fully unravel the characteristics of these transiently appearing structures, mainly due to the lack of the spatial resolution of optical microscopy. Scanning electron microscopy (SEM), in principle, enables the direct visualization of nanoscopic architectures of the tethers and slings (see below). However, it has been difficult to chemically fix and preserve the thin and elastic tethers and slings,^52^ which is the prerequisite for SEM measurements. We previously reported the chemical fixation of KG1a cells rolled over the rh E-selectin surface inside a fluidic chamber, which allowed to preserve transient cell architectures such as elongated microvilli but not tethers and slings.^2^ We modified the protocol to avoid the formation of air bubbles that occurs at higher shear stresses during the perfusion of the fixatives used to chemically preserve structures on rolled cells inside the fluidic chamber. Immunofluorescence images of CD44 (labeled by AF-647 dye-conjugated antibody) on fixed KG1a cells rolled over the CHO-E monolayer exhibited the formation of tethers (Figure 4e). The superimposed bright-field and fluorescence image showed the tethers were anchored to the CHO-E cells (Figure 4e). The average length of the tethers (13 μm) and slings (18 μm) matches the mean length of tethers observed in live-cell imaging (Figure 4c). Confocal fluorescence microscopy imaging of fixed KG1a cells rolled over the CHO-E monolayer also revealed intact tethers and slings (Figure S6). The statistical analysis showed that the mean lengths of the tethers (17 μm) and slings (19 μm) captured by the confocal microscopy experiment are consistent with those determined for the live KGa1 cells (Figure 4c). In addition, the length distribution of the tethers and slings is very similar in both live and fixed cells, including the maximum values. Together, these results suggest that the morphology of the rolled cells at a scale larger than the diffraction-limited size (∼300 nm in our experiment) has been preserved during the fixation in the fluidic chamber.

### Electron Microscopy Characterization of KG1a Rolling over a CHO-E Monolayer

KG1a cells for SEM imaging studies were prepared by treating the chemically fixed cells with osmium tetroxide followed by dehydration by ethanol and drying by a critical point dryer (see the Experimental Section for details). We found that standard cell fixation steps for SEM imaging incurred cell damage, including the loss of fragile structures such as tethers and slings. Thus, we developed a fixation protocol that enables us to preserve fragile structures. This includes (1) lowering the concentration of glutaraldehyde from 2.5 to 2%, (2) exchanging all solutions in the chamber in a dropwise manner, and (3) slower gas exchange uptake and slower speed of gas output during the treatment with the critical point dryer.

**Figure 5:**
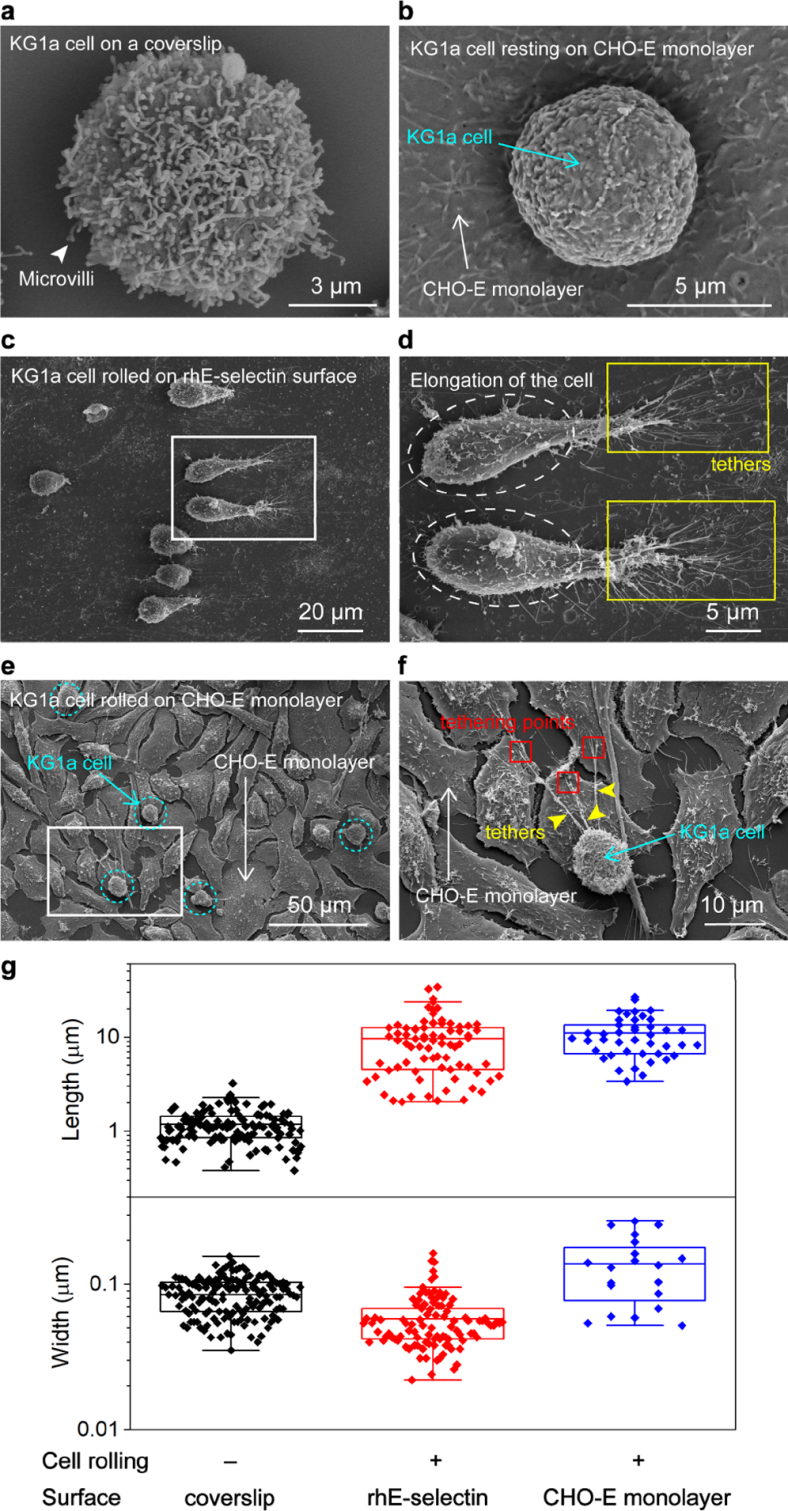
Scanning electron microscopy (SEM) characterization of nanoscopic morphology of KG1a cells during the cell rolling over the CHO-E monolayer. SEM images of KG1a cells deposited on the (a) coverslip and (b) CHO-E monolayer. (c) SEM image of KG1a cells rolled on a rh E-selectin-coated surface (0.2 μg mL^–1^) at a shear stress of 1 dyne cm^–2^. (d) Enlarged view of the area highlighted by the white box in panel (c). Tethers formed during the cell rolling are highlighted by yellow boxes. Elongation of the KG1a cells is highlighted by ellipses. (e) SEM image of KG1a cells rolled on the CHO-E monolayer at a shear stress of 2 dyne cm^–2^. KG1a cells are highlighted by circles. (f) Enlarged view of the area highlighted by the box in panel (e). Tethers formed during the cell rolling are highlighted by arrowheads. Tethering points are highlighted by red squares. (g) Box plots showing the length (top) and width (bottom) of the tethers and slings captured by SEM measurements.

SEM images of KG1a cells at the resting state (i.e., cells deposited on a coverslip) showed a spherical shape of the cell with short microvilli protruding structures (Figures 5a and S7). The mean length of the microvilli was estimated to be 1.1 μm. SEM images of KG1a cells resting on the CHO-E monolayer (i.e., cells were deposited on the CHO-E monolayer without external shear stress) showed the spherical shape of the cells with the surface morphology distinct from that of the KG1a cells deposited on a coverslip. The microvilli protruding structures are less obvious on the KG1a cell resting of the CHO-E monolayer (Figure 5b). Complex cell-to-cell interactions could affect the surface morphology of the KG1a cells.^54,55^

We first investigated the effects of cell rolling on its morphology changes by capturing SEM images of KG1a cells rolled over rh E-selectin (surface density of rh E-selectin = 3.6 molecules μm^–2^, Figures 5c,d and S8). The SEM image revealed its intrinsic morphological details of slings and tethers elongation upon cell rolling (Figures 5d and S9). We found that the optimized fixation protocol for the SEM imaging resulted in similar mean lengths of the tethers and slings captured by the SEM (9.6 μm, Figure 5g) and fluorescence microscopy imaging experiments (Figure 4c),^3^ suggesting that the fragile transient structures such as tethers and slings were well-preserved during the fixation of the cells for the SEM characterization. We noticed that very long tethers and slings (>30 μm) are often absent in the SEM images, indicating the loss of those infrequently appearing transient structures during the fixation. Overall, our results suggest that the nanoscopic morphological features of the cells appearing transiently during cell rolling are well-preserved during the treatments for preparing the SEM samples, except for very long tethers and slings that exceed the length of 30 μm.

Although the formation of the tethers on neutrophils rolling over P-selectin has been captured by SEM,^56^ the reported length of the tethers (3–8 μm) is much shorter than that of the KG1a cell rolling over E-selectin. Interestingly, we observed a morphology change of the cell body from spherical to elongated shape upon the rolling over rh E-selectin (Figures 5d and S9). The mean aspect ratio (length of the cell along the long axis divided by the length of the cell along the short axis) of the rolled cells determined by the SEM images (1.8:1) was much larger than that obtained for the resting cells (1.0), indicating that the shear stress exerted to the cells caused the elongation of cells.

We next investigated the effects of the rolling of KG1a cells over the CHO-E monolayer on its morphology (Figures 5e and S10). The SEM images clearly showed that the formed tethers are anchored to the CHO-E monolayer (Figures 5f and S11). While the mean length of the tethers (11 μm) is similar to that of the KG1a cell rolled over rh E-selectin (Figure 5g), we observed much less elongation of the rolled cells (aspect ratio of 1.0, Figures 5f and S11). The finding may indicate a smaller effective shear force exerted on the KG1a cells rolling over the CHO-E monolayer compared with those rolling over rh E-selectin because of the elastic property of the cells. The cell-rolling assay demonstrated a slight dependence of the rolling velocity of KG1a cells to the shear stress on the CHO-E monolayer (Figure 3e) that contrasts to the linear dependence of the rolling velocity to the shear stress observed in the KG1a cells rolling over rh E selection,^2^ supporting this hypothesis. Previous studies suggested that stable shear-resistant cell rolling requires cellular properties by comparing the rolling of cells and microspheres coated by selectin ligands over P-selectin^57^ although details of the cellular contribution to the stable cell rolling remained unclear. The results of the SEM imaging experiment indicate that our new method could provide a powerful means to investigate the role of nanoscopic cellular architecture on the rolling of the cells.

The high spatial resolution of SEM enabled us to obtain not only the length of the tethers but also their width. The mean widths of the single tethers formed during the rolling of KG1a cells on rh E-selectin and the CHO-E monolayer were estimated to be 0.088 and 0.18 μm, respectively (Figure 5g). These widths are close to the mean widths of the microvilli observed on the surface of the resting KG1a cells (0.097 μm, Figure 5g). We also found that the width of the tethers does not depend significantly on their length (Figure S12). We previously showed that the tethers are formed from single microvilli (i.e., tethers are formed via the elongation of the microvilli).^3^ These results could be interpreted by cell plasma membrane flowing into the tethers, which was indicated in previous studies.^58,59^

We showed that the protocol for the SEM imaging of the rolled cells that we developed in this study allowed us to preserve and visualize cell body intrinsic details as well as microvilli and structural details of tethers and slings at a high resolution under the condition close to in vivo HSPC rolling (i.e., cell rolling over the CHO-E monolayer). The SEM images provide new detailed insight into the dramatic morphological changes that occur during cell rolling and migration, which have not been reported previously, and open new opportunities to unravel complicated molecular and cellular mechanisms of not only HSPC homing but also cell migration processes as a whole.

## Conclusions

In this study, we developed a new microfluidics-based experimental platform that enabled us to characterize the spatiotemporal details of the cell-rolling migration occurring under conditions close to the in vivo environment using the endothelium-like cell monolayer. While the live-cell fluorescence imaging allowed us to characterize the temporal dynamics of cell rolling, scanning electron microscopy characterization captured the nanoscopic morphology of the migrating cells that transiently appeared during cell rolling.

The developed experimental method allows for the efficient collection of visual and analytical information on spatial nanoscopic architectures of protein–protein interactions occurring at the cellular interface. Using such an approach, characterization of the nanoscopic interactions between selectins expressed on the endothelium layer and their ligands expressed on rolling cells could be captured, providing valuable insight into true in vivo processes. Beyond the interactions studied here, this method will open avenues for better understanding, from a nanoscopic perspective, cell motility, and cell migration beyond tethering and rolling such as integrin activation and firm adhesion as well as intercellular dynamics and commitment to transendothelial migration via paracellular or transcellular routes. Focus on how the microenvironment through to stimuli, such as cytokines and chemokines, determines subsequent effects on cellular plasticity, cytoskeletal rearrangements, and morphology could also be explored.

## Supporting Information Available

Bright-field images of cultured CHO-E cells in the fluidic chamber (Figure S1); viability assay of the CHO-E monolayer in the fluidic chamber (Figure S2); CHO-E monolayer cell culture before and after shear (Figure S3); CHO-K monolayer not expressing E-selectin (Figure S4); examples of long tether and slings on fixed KG1a rolled on the CHO-E monolayer (Figure S5); 3D confocal fluorescence image of fixed KG1a rolled on the CHO-E monolayer (Figure S6); SEM image of nonrolled KG1a cells on a coverslip (Figure S7); SEM image of rolled KG1a cells on the rh E-selectin-coated surface (Figure S8); SEM enlarged view of rolled KG1a cells on the rh E-selectin-coated surface (Figure S9); SEM image of KG1a cells rolled on the CHO-E monolayer (Figure S10); SEM enlarged view of rolled KG1a cells on the CHO-E monolayer (Figure S11); and correlation between the length and width of the tethers and slings formed on rolled KG1a on various surfaces (Figure S12) (PDF)
Time-lapse bright-field microscopy images of KG1a cells rolling over the CHO-E monolayer (MP4)
Time-lapse bright-field microscopy images of KG1a cells rolling over the CHO-E monolayer in the presence of 10 mM EDTA (MP4)
Time-lapse bright-field microscopy images of KG1a cells rolling over the CHO-K monolayer (MP4)

## References

[ref1] Jagannathan-Bogdan M.; Zon L. I. Hematopoiesis. Development 2013, 140, 2463–2467. 10.1242/dev.083147. 23715539 PMC3666375

[ref2] AbuZineh K.; Joudeh L. I.; Al Alwan B.; Hamdan S. M.; Merzaban J. S.; Habuchi S. Microfluidics-based super-resolution microscopy enables nanoscopic characterization of blood stem cell rolling. Sci. Adv. 2018, 4, eaat5304 10.1126/sciadv.aat5304. 30035228 PMC6051739

[ref3] Al Alwan B.; AbuZineh K.; Nozue S.; Rakhmatulina A.; Aldehaiman M.; Al-Amoodi A. S.; Serag M. F.; Aleisa F. A.; Merzaban J. S.; Habuchi S. Single-molecule imaging and microfluidic platform reveal molecular mechanisms of leukemic cell rolling. Commun. Biol. 2021, 4, 868 10.1038/s42003-021-02398-2. 34262131 PMC8280113

[ref4] Sackstein R. The bone marrow is akin to skin: HCELL and the biology of hematopoietic stem cell homing. J. Invest. Dermatol. Symp. Proc. 2004, 9, 215–223. 10.1016/S0022-202X(15)53011-X. 15369216

[ref5] Veerman K. M.; Williams M. J.; Uchimura K.; Singer M. S.; Merzaban J. S.; Naus S.; Carlow D. A.; Owen P.; Rivera-Nieves J.; Rosen S. D.; Ziltener H. J. Interaction of the selectin ligand PSGL-1 with chemokines CCL21 and CCL19 facilitates efficient homing of T cells to secondary lymphoid organs. Nat. Immunol. 2007, 8, 532–539. 10.1038/ni1456. 17401367

[ref6] Suarez-Alvarez B.; Lopez-Vazquez A.; Lopez-Larrea C. Mobilization and Homing of Hematopoietic Stem Cells. In Stem Cell Transplantation; LopezLarrea C.; LopezVazquez A.; SuarezAlvarez B., Eds.; Springer-Verlag Berlin: Berlin, 2012; Vol. 741, pp 152–170. 10.1007/978-1-4614-2098-9_11 22457109

[ref7] Pereira J. L.; Cavaco P.; da Silva R. C.; Pacheco-Leyva I.; Mereiter S.; Pinto R.; Reis C. A.; Dos Santos N. R. P-selectin glycoprotein ligand 1 promotes T cell lymphoma development and dissemination. Transl. Oncol. 2021, 14, 101125 10.1016/j.tranon.2021.101125. 34090013 PMC8188565

[ref8] Crane G. M.; Jeffery E.; Morrison S. J. Adult haematopoietic stem cell niches. Nat. Rev. Immunol. 2017, 17, 573–590. 10.1038/nri.2017.53. 28604734

[ref9] McEver R. P. Selectins: initiators of leucocyte adhesion and signalling at the vascular wall. Cardiovasc. Res. 2015, 107, 331–339. 10.1093/cvr/cvv154. 25994174 PMC4592324

[ref10] Vestweber D. How leukocytes cross the vascular endothelium. Nat. Rev. Immunol. 2015, 15, 692–704. 10.1038/nri3908. 26471775

[ref11] Sundd P.; Gutierrez E.; Koltsova E. K.; Kuwano Y.; Fukuda S.; Pospieszalska M. K.; Groisman A.; Ley K. ‘Slings’ enable neutrophil rolling at high shear. Nature 2012, 488, 399–403. 10.1038/nature11248. 22763437 PMC3433404

[ref12] Li W. W.; Mao S. F.; Khan M.; Zhang Q.; Huang Q. S.; Feng S.; Lin J. M. Responses of Cellular Adhesion Strength and Stiffness to Fluid Shear Stress during Tumor Cell Rolling Motion. ACS Sens. 2019, 4, 1710–1715. 10.1021/acssensors.9b00678. 31094503

[ref13] Simone G.; Perozziello G.; Battista E.; De Angelis F.; Candeloro P.; Gentile F.; Malara N.; Manz A.; Carbone E.; Netti P.; Di Fabrizio E. Cell rolling and adhesion on surfaces in shear flow. A model for an antibody-based microfluidic screening system. Microelectron. Eng. 2012, 98, 668–671. 10.1016/j.mee.2012.07.008.

[ref14] Ali A. J.; Abuelela A. F.; Merzaban J. S. An Analysis of Trafficking Receptors Show that CD44 and P-Selectin Glycoprotein Ligand-1 Collectively Control the Migration of Activated Human T-Cells. Front. Immunol. 2017, 8, 492 10.3389/fimmu.2017.00492. 28515724 PMC5413510

[ref15] Carlow D. A.; Tra M. C.; Ziltener H. J. A cell-extrinsic ligand acquired by activated T cells in lymph node can bridge L-selectin and P-selectin. PLoS One 2018, 13, e0205685 10.1371/journal.pone.0205685. 30379850 PMC6209203

[ref16] Matsumoto M.; Miyasaka M.; Hirata T. P-Selectin Glycoprotein Ligand-1 Negatively Regulates T-Cell Immune Responses. J. Immunol. 2009, 183, 7204–7211. 10.4049/jimmunol.0902173. 19890058

[ref17] Birbrair A.; Frenette P. S. Niche heterogeneity in the bone marrow. Ann. N. Y. Acad. Sci. 2016, 1370, 82–96. 10.1111/nyas.13016. 27015419 PMC4938003

[ref18] Sipkins D. A.; Wei X. B.; Wu J. W.; Runnels J. M.; Cote D.; Means T. K.; Luster A. D.; Scadden D. T.; Lin C. P. In vivo imaging of specialized bone marrow endothelial microdomains for tumour engraftment. Nature 2005, 435, 969–973. 10.1038/nature03703. 15959517 PMC2570168

[ref19] Narasipura S. D.; Wojciechowski J. C.; Charles N.; Liesveld J. L.; King M. R. P-selectin-coated microtube for enrichment of CD34(+) hematopoietic stem and progenitor cells from human bone marrow Hematology. Clin. Chem. 2008, 54, 77–85. 10.1373/clinchem.2007.089896. 18024531

[ref20] Al-Amoodi A. S.; Li Y. Y.; Al-Ghuneim A.; Allehaibi H.; Isaioglou I.; Esau L. E.; AbuSamra D. B.; Merzaban J. S. Refining the migration and engraftment of short-term and long-term HSCs by enhancing homing-specific adhesion mechanisms. Blood Adv. 2022, 6, 4373–4391. 10.1182/bloodadvances.2022007465. 35764498 PMC9636332

[ref21] AbuSamra D. B.; Aleisa F. A.; Al-Amoodi A. S.; Ahmed H. M. J.; Chin C. J.; Abuelela A. F.; Bergam P.; Sougrat R.; Merzaban J. S. Not just a marker: CD34 on human hematopoietic stem/progenitor cells dominates vascular selectin binding along with CD44. Blood Adv. 2017, 1, 2799–2816. 10.1182/bloodadvances.2017004317. 29296932 PMC5745127

[ref22] Abadier M.; Pramod A. B.; McArdle S.; Marki A.; Fan Z. C.; Gutierrez E.; Groisman A.; Ley K. Effector and Regulatory T Cells Roll at High Shear Stress by Inducible Tether and Sling Formation. Cell Rep. 2017, 21, 3885–3899. 10.1016/j.celrep.2017.11.099. 29281835 PMC5786164

[ref23] Ley K.; Laudanna C.; Cybulsky M. I.; Nourshargh S. Getting to the site of inflammation: the leukocyte adhesion cascade updated. Nat. Rev. Immunol. 2007, 7, 678–689. 10.1038/nri2156. 17717539

[ref24] McEver R. P.; Zhu C. Rolling Cell Adhesion. Annu. Rev. Cell Dev. Biol. 2010, 26, 363–396. 10.1146/annurev.cellbio.042308.113238. 19575676 PMC3557855

[ref25] Fan Z. C.; McArdle S.; Marki A.; Mikulski Z.; Gutierrez E.; Engelhardt B.; Deutsch U.; Ginsberg M.; Groisman A.; Ley K. Neutrophil recruitment limited by high-affinity bent beta(2) integrin binding ligand in cis. Nat. Commun. 2016, 7, 12658 10.1038/ncomms12658. 27578049 PMC5013657

[ref26] Duchamp M.; Dahoun T.; Vaillier C.; Arnaud M.; Bobisse S.; Coukos G.; Harari A.; Renaud P. Microfluidic device performing on flow study of serial cell-cell interactions of two cell populations. RSC Adv. 2019, 9, 41066–41073. 10.1039/C9RA09504G. 35540074 PMC9076435

[ref27] Marki A.; Gutierrez E.; Mikulski Z.; Groisman A.; Ley K. Microfluidics-based side view flow chamber reveals tether-to-sling transition in rolling neutrophils. Sci. Rep. 2016, 6, 28870 10.1038/srep28870. 27357741 PMC4928115

[ref28] Cugno A.; Marki A.; Ley K. Biomechanics of Neutrophil Tethers. Life 2021, 11, 515 10.3390/life11060515. 34073130 PMC8230032

[ref29] AbuElela A. F.; Al-Amoodi A. S.; Ali A. J.; Merzaban J. S. Fluorescent Multiplex Cell Rolling Assay: Simultaneous Capturing up to Seven Samples in Real-Time Using Spectral Confocal Microscopy. Anal. Chem. 2020, 92, 6200–6206. 10.1021/acs.analchem.9b04549. 32264668

[ref30] Aleisa F. A.; Sakashita K.; Lee J. M.; AbuSamra D. B.; Al Alwan B.; Nozue S.; Tehseen M.; Hamdan S. M.; Habuchi S.; Kusakabe T.; Merzaban J. S. Functional binding of E-selectin to its ligands is enhanced by structural features beyond its lectin domain. J. Biol. Chem. 2020, 295, 3719–3733. 10.1074/jbc.RA119.010910. 31949047 PMC7076219

[ref31] Kotha S. S.; Hayes B. J.; Phong K. T.; Redd M. A.; Bomsztyk K.; Ramakrishnan A.; Torok-Storb B.; Zheng Y. Engineering a multicellular vascular niche to model hematopoietic cell trafficking. Stem Cell Res. Ther. 2018, 9, 1–14. 10.1186/s13287-018-0808-2. 29566751 PMC5865379

[ref32] Dabagh M.; Gounley J.; Randles A. Localization of Rolling and Firm-Adhesive Interactions Between Circulating Tumor Cells and the Microvasculature Wall. Cell. Mol. Bioeng. 2020, 13, 141–154. 10.1007/s12195-020-00610-7. 32175027 PMC7048902

[ref33] Robert P.; Touchard D.; Bongrand P.; Pierres A. Biophysical description of multiple events contributing blood leukocyte arrest on endothelium. Front. Immunol. 2013, 4, 108 10.3389/fimmu.2013.00108. 23750158 PMC3654224

[ref34] Kwon S.; Kurmashev A.; Lee M. S.; Kang J. H. An inflammatory vascular endothelium-mimicking microfluidic device to enable leukocyte rolling and adhesion for rapid infection diagnosis. Biosens. Bioelectron. 2020, 168, 112558 10.1016/j.bios.2020.112558. 32911451

[ref35] Ley K.; Bullard D. C.; Arbones M. L.; Bosse R.; Vestweber D.; Tedder T. F.; Beaudet A. L. Sequential contribution of L-selectin and P = selectin to leukocyte rolling in-vivo. J. Exp. Med. 1995, 181, 669–675. 10.1084/jem.181.2.669. 7530761 PMC2191869

[ref36] Itkin T.; Gur-Cohen S.; Spencer J. A.; Schajnovitz A.; Ramasamy S. K.; Kusumbe A. P.; Ledergor G.; Jung Y.; Milo I.; Poulos M. G.; Kalinkovich A.; Ludin A.; et al. Distinct bone marrow blood vessels differentially regulate haematopoiesis. Nature 2016, 532, 323–328. 10.1038/nature17624. 27074509 PMC6450701

[ref37] Mendez-Ferrer S.; Frenette P. S. Hematopoietic stem cell trafficking: regulated adhesion and attraction to bone marrow microenvironment. Ann. N. Y. Acad. Sci. 2007, 1116, 392–413. 10.1196/annals.1402.086. 18083941

[ref38] Wojciechowski J. C.; Narasipura S. D.; Charles N.; Mickelsen D.; Rana K.; Blair M. L.; King M. R. Capture and enrichment of CD34-positive haematopoietic stem and progenitor cells from blood circulation using P-selectin in an implantable device. Br. J. Haematol. 2008, 140, 673–681. 10.1111/j.1365-2141.2007.06967.x. 18218048 PMC2268974

[ref39] Sackstein R.; Merzaban J. S.; Cain D. W.; Dagia N. M.; Spencer J. A.; Lin C. P.; Wohlgemuth R. Ex vivo glycan engineering of CD44 programs human multipotent mesenchymal stromal cell trafficking to bone. Nat. Med. 2008, 14, 181–187. 10.1038/nm1703. 18193058

[ref40] Herisson F.; Frodermann V.; Courties G.; Rohde D.; Sun Y.; Vandoorne K.; Wojtkiewicz G. R.; Masson G. S.; Vinegoni C.; Kim J.; Kim D. E.; Weissleder R.; Swirski F. K.; Moskowitz M. A.; Nahrendorf M. Direct vascular channels connect skull bone marrow and the brain surface enabling myeloid cell migration. Nat. Neurosci. 2018, 21, 1209–1217. 10.1038/s41593-018-0213-2. 30150661 PMC6148759

[ref41] AbuSamra D. B.; Al-Kilani A.; Hamdan S. M.; Sakashita K.; Gadhoum S. Z.; Merzaban J. S. Quantitative Characterization of E-selectin Interaction with Native CD44 and P-selectin Glycoprotein Ligand-1 (PSGL-1) Using a Real Time Immunoprecipitation-based Binding Assay. J. Biol. Chem. 2015, 290, 21213–21230. 10.1074/jbc.M114.629451. 26124272 PMC4571854

[ref42] Abadi M.; Serag M. F.; Habuchi S. Entangled polymer dynamics beyond reptation. Nat. Commun. 2018, 9, 5098 10.1038/s41467-018-07546-7. 30504765 PMC6269522

[ref43] Piwoński H.; Michinobu T.; Habuchi S. Controlling photophysical properties of ultrasmall conjugated polymer nanoparticles through polymer chain packing. Nat. Commun. 2017, 8, 15256 10.1038/ncomms15256. 28508857 PMC5440812

[ref44] Serag M. F.; Abadi M.; Habuchi S. Single-molecule diffusion and conformational dynamics by spatial integration of temporal fluctuations. Nat. Commun. 2014, 5, 5123 10.1038/ncomms6123. 25283876 PMC4205855

[ref45] Huang R. B.; Eniola-Adefeso O. Shear Stress Modulation of IL-1 beta-Induced E-Selectin Expression in Human Endothelial Cells. PLoS One 2012, 7, e31874 10.1371/journal.pone.0031874. 22384091 PMC3286450

[ref46] Merzaban J. S.; Burdick M. M.; Gadhoum S. Z.; Dagia N. M.; Chu J. T.; Fuhlbrigge R. C.; Sackstein R. Analysis of glycoprotein E-selectin ligands on human and mouse marrow cells enriched for hematopoietic stem/progenitor cells. Blood 2011, 118, 1774–1783. 10.1182/blood-2010-11-320705. 21659548 PMC3158712

[ref47] Fay M. E.; Myers D. R.; Kumar A.; Turbyfield C. T.; Byler R.; Crawford K.; Mannino R. G.; Laohapant A.; Tyburski E. A.; Sakurai Y.; Rosenbluth M. J.; Switz N. A.; Sulchek T. A.; Graham M. D.; Lam W. A. Cellular softening mediates leukocyte demargination and trafficking, thereby increasing clinical blood counts. Proc. Natl. Acad. Sci. U.S.A. 2016, 113, 1987–1992. 10.1073/pnas.1508920113. 26858400 PMC4776450

[ref48] Hanley W. D.; Wirtz D.; Konstantopoulos K. Distinct kinetic and mechanical properties govern selectin-leukocyte interactions. J. Cell Sci. 2004, 117, 2503–2511. 10.1242/jcs.01088. 15159451

[ref49] Li I. T. S.; Ha T.; Chemla Y. R. Mapping cell surface adhesion by rotation tracking and adhesion footprinting. Sci. Rep. 2017, 7, 44502 10.1038/srep44502. 28290531 PMC5349612

[ref50] Marki A.; Buscher K.; Lorenzini C.; Meyer M.; Saigusa R.; Fan Z.; Yeh Y.-T.; Hartmann N.; Dan J. M.; Kiosses W. B.; et al. Elongated neutrophil-derived structures are blood-borne microparticles formed by rolling neutrophils during sepsis. J. Exp. Med. 2021, 218, e20200551 10.1084/jem.20200551. 33275138 PMC7721910

[ref51] Murphy D. B. Fundamentals of Light Microscopy and Electronic Imaging; John Wiley & Sons, 2002.

[ref52] Marki A.; Buscher K.; Mikulski Z.; Pries A.; Ley K. Rolling neutrophils form tethers and slings under physiologic conditions in vivo. J. Leukocyte Biol. 2018, 103, 67–70. 10.1189/jlb.1AB0617-230R. 28821572 PMC6347012

[ref53] Ghosh S.; Di Bartolo V.; Tubul L.; Shimoni E.; Kartvelishvily E.; Dadosh T.; Feigelson S. W.; Alon R.; Alcover A.; Haran G. ERM-Dependent Assembly of T Cell Receptor Signaling and Co-stimulatory Molecules on Microvilli prior to Activation. Cell Rep. 2020, 30, 3434–3447. 10.1016/j.celrep.2020.02.069. 32160548

[ref54] Armingol E.; Officer A.; Harismendy O.; Lewis N. E. Deciphering cell-cell interactions and communication from gene expression. Nat. Rev. Genet. 2021, 22, 71–88. 10.1038/s41576-020-00292-x. 33168968 PMC7649713

[ref55] Bich L.; Pradeu T.; Moreau J. F. Understanding Multicellularity: The Functional Organization of the Intercellular Space. Front. Physiol. 2019, 10, 1170 10.3389/fphys.2019.01170. 31620013 PMC6759637

[ref56] Ramachandran V.; Williams M.; Yago T.; Schmidtke D. W.; McEver R. P. Dynamic alterations of membrane tethers stabilize leukocyte rolling on P-selectin. Proc. Natl. Acad. Sci. U.S.A. 2004, 101, 13519–13524. 10.1073/pnas.0403608101. 15353601 PMC518789

[ref57] Yago T.; Leppanen A.; Qiu H. Y.; Marcus W. D.; Nollert M. U.; Zhu C.; Cummings R. D.; McEver R. P. Distinct molecular and cellular contributions to stabilizing selectin-mediated rolling under flow. J. Cell Biol. 2002, 158, 787–799. 10.1083/jcb.200204041. 12177042 PMC2174028

[ref58] Evans E.; Yeung A. Hidden dynamics in rapid changes of bilayer shape. Chem. Phys. Lipids 1994, 73, 39–56. 10.1016/0009-3084(94)90173-2.

[ref59] Pospieszalska M. K.; Ley K. Dynamics of Microvillus Extension and Tether Formation in Rolling Leukocytes. Cell. Mol. Bioeng. 2009, 2, 207–217. 10.1007/s12195-009-0063-9. 20046963 PMC2747329

