# Supplementary material for: Nanoscopic Characterization of Cell Migration under Flow Using Optical and Electron Microscopy
Source: Anal Chem. 2023 Jan 10;95(3):1958–66. doi: 10.1021/acs.analchem.2c04222 (PMC9878504; doi:10.1021/acs.analchem.2c04222)
Supplement: Supplementary file 1 — ac2c04222_si_001.pdf [file ac2c04222_si_001.pdf]

Supporting Information for

Nanoscopic Characterization of Cell Migration under Flow Using Optical and Electron Microscopy

*Abdullah Alghamdi‡, Amar Tamra‡, Aigerim Rakhmatulina‡, Shuho Nozue, Asma S. Al-Amoodi, Mansour M. Aldehaiman, Ioannis Isaioglou, Jasmeen S. Merzaban, Satoshi Habuchi**

Biological and Environmental Science and Engineering Division, King Abdullah University of Science and Technology, Thuwal 23955-6900, Saudi Arabia

‡These authors contributed equally.

*Correspondence should be addressed to S.H. (E-mail: Satoshi.Habuchi@kaust.edu.sa).

Contents

Supporting Figures: Figures S1–S12

Captions for Supporting Videos: Video S1-S3

Supporting Figures

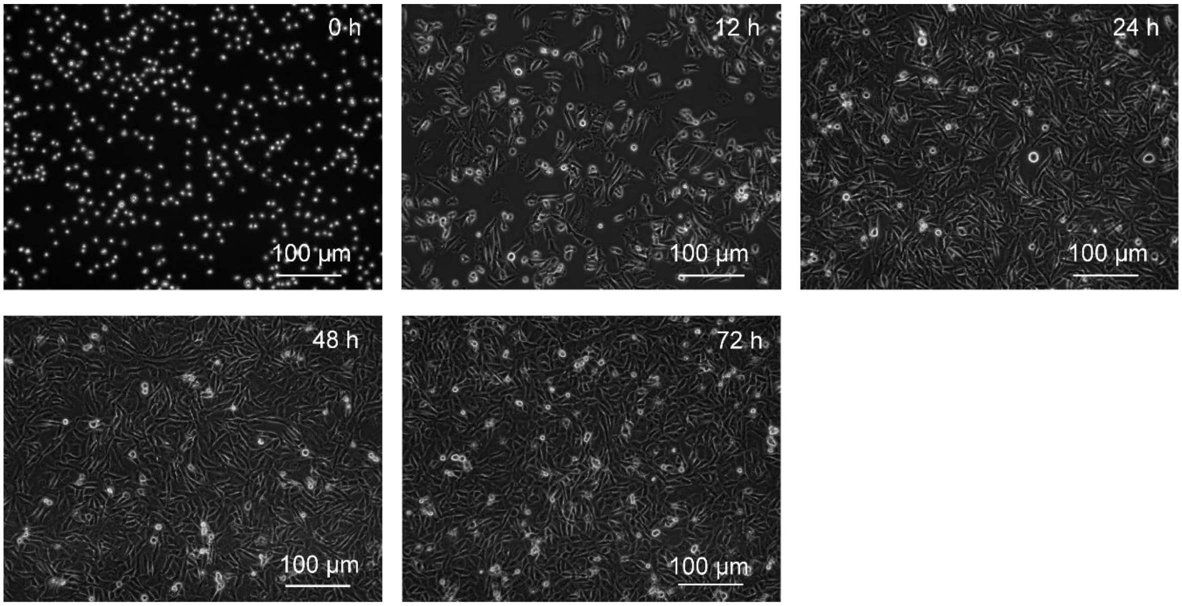

Figure S1. Bright-field images of cultured CHO-E cells in the fluidic chamber captured at different time points. The cells were seeded over a glass-bottom fibronectin-coated surface.

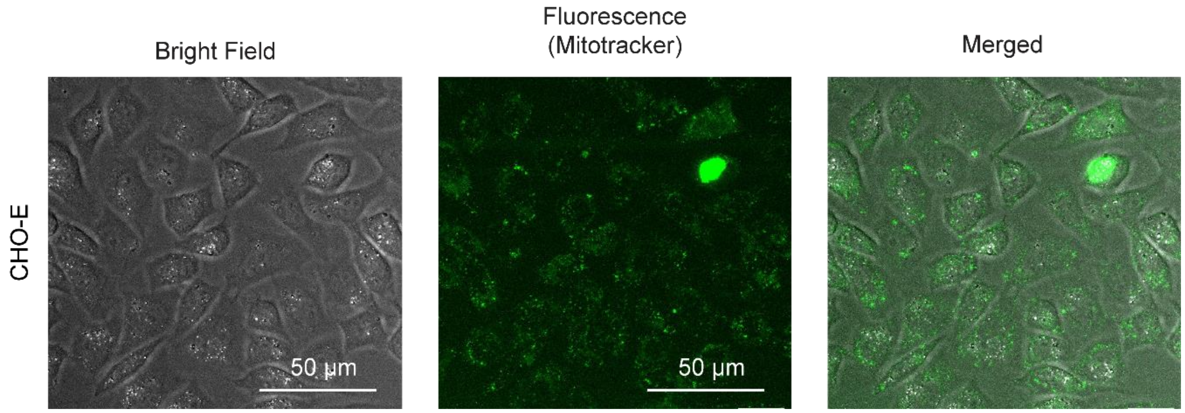

Figure S2. Viability assay of CHO-E monolayer cultured in the fluidic chamber. Bright filed (left) and fluorescence (center) images captured at the same sample area. The CHO-E cells were stained with Mito-tracker deep red that shows fluorescence only in the dead cells. The right panel shows merged image.

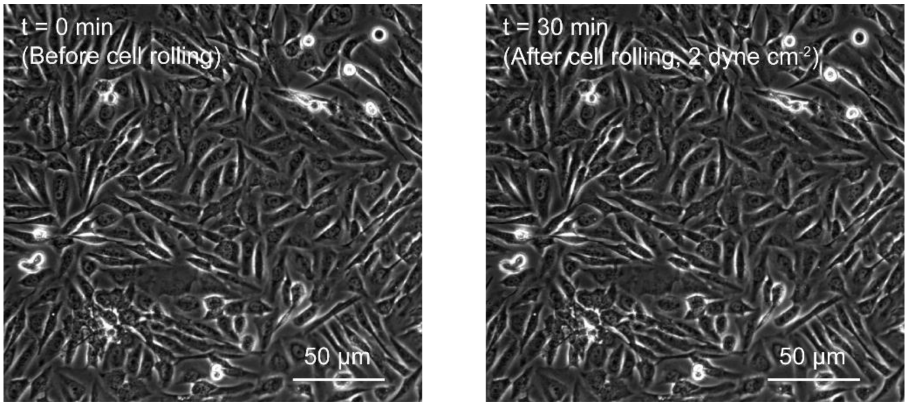

Figure S3. Bright field images of the CHO-E monolayer cell culture captured before (left) and after (right) perfusing rolling buffer at the shear stress of 2 dyne cm^{-2} for 10 min.

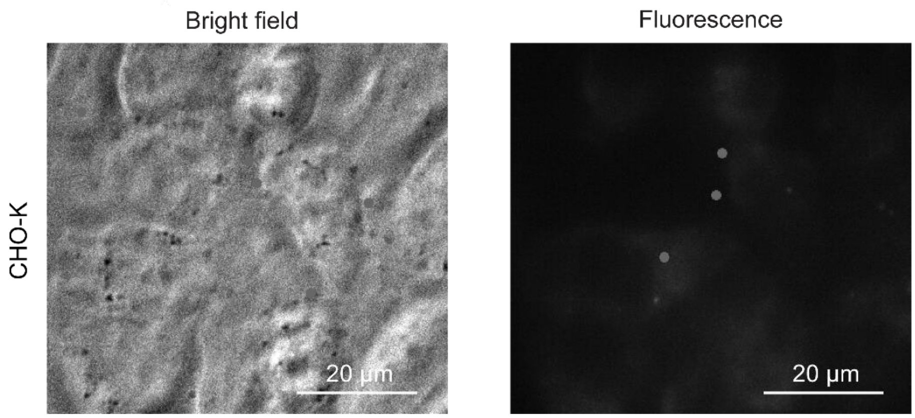

Figure S4. Bright field and fluorescence images of fixed and stained CHO-K cells (that do not express human E-selectin). E-selectin surface receptors were immunolabeled using primary antibodies and secondary AF-647 conjugated antibodies.

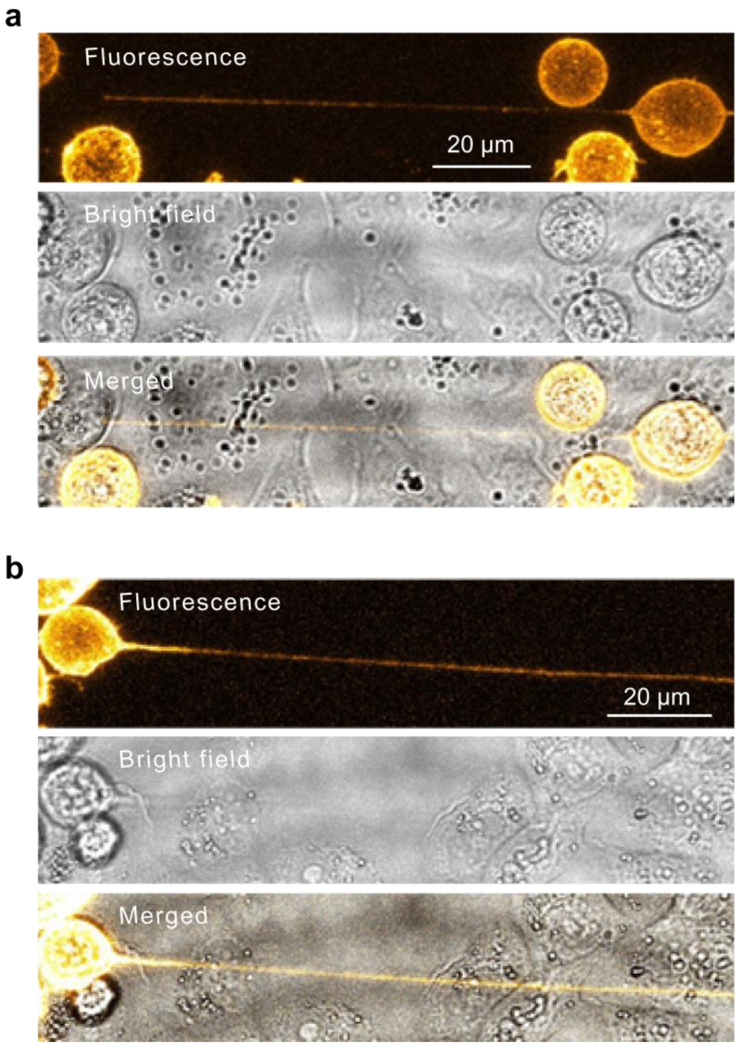

Figure S5. Examples of (a) long tether and (b) long slings on KG1a cells rolled on the CHO-E monolayer. Fluorescence (top, immunolabeled by AF-647 dye-conjugated anti-CD44 antibody) and bright field (middle) images captured during the KG1a cells rolling on the CHO-E monolayer at the shear stress of 2 dyne cm^{-2}.

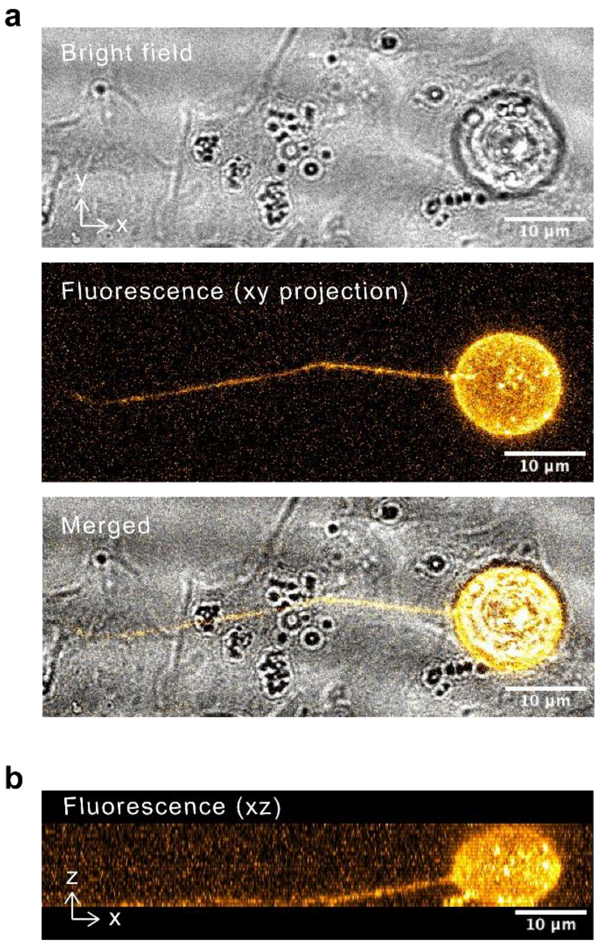

Figure S6. KG1a cell fixed after rolled on CHO-E monolayer at a shear stress of 2 dyne cm^{-2}. (a) Bright field (top) and fluorescence (middle, mmunolabeled by AF-647 dye-conjugated anti-CD44 antibody) images of rolled KG1a cells. The fluorescence image shows 2D projection of the 3D image on the xy plane captured using confocal microscopy. (b) Confocal image of the rolled KG1a cell (2D projection of the 3D image on the xz plane).

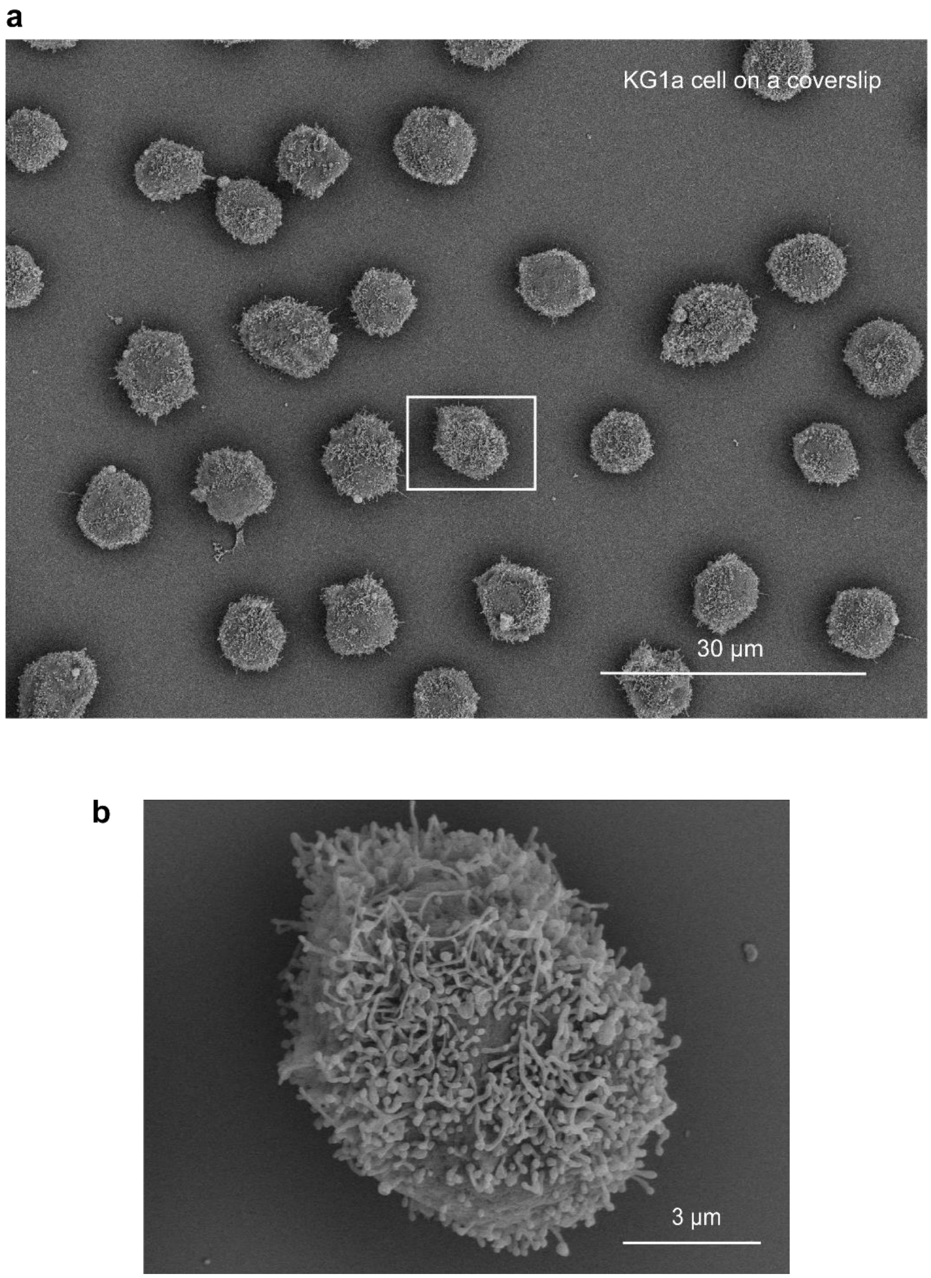

Figure S7. (a) SEM image of KG1a cells deposited on a coverslip. (b) Enlarged view of the area highlighted by the white box in (a).

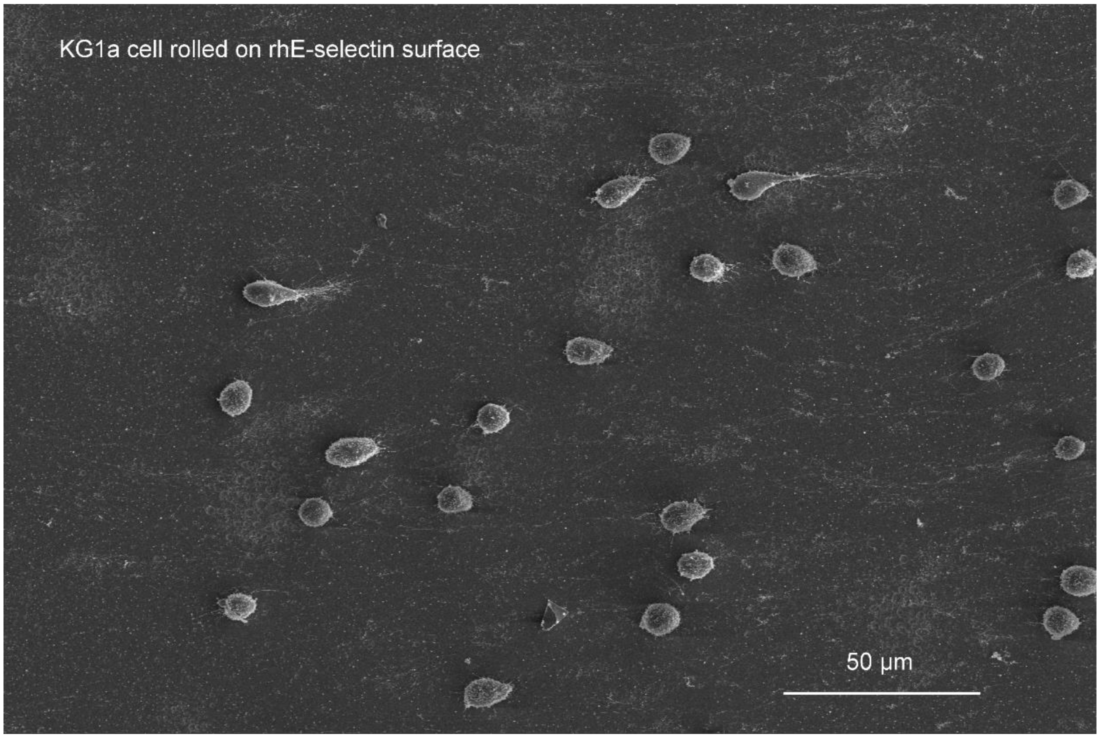

Figure S8. SEM image of KG1a cells rolled on a rhE-selectin coated surface (2μg ml^{-1}) at the shear stress of 1 dyne cm^{-2}.

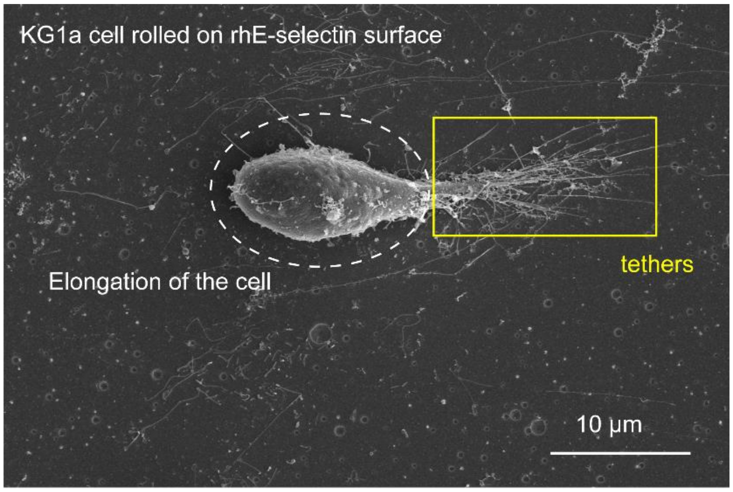

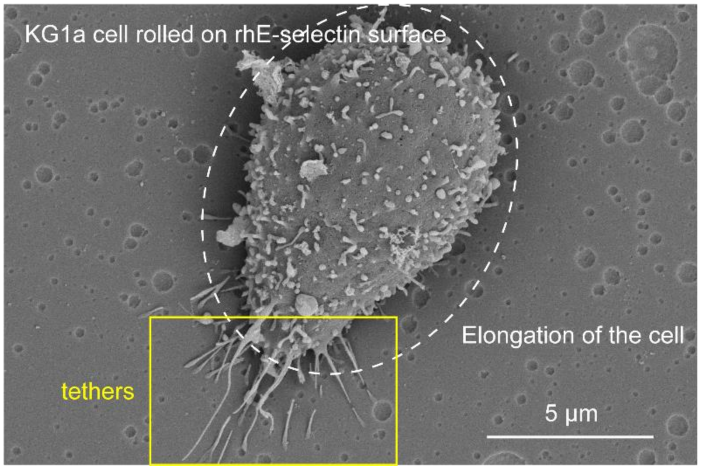

Figure S9. Enlarged view of KG1a cells rolled on a rhE-selectin coated surface (2μg ml^{-1}) at the shear stress of 1 dyne cm^{-2}. Tethers formed during the cell rolling are highlighted by yellow boxes. Elongation of the KG1a cells are highlighted by ellipses.

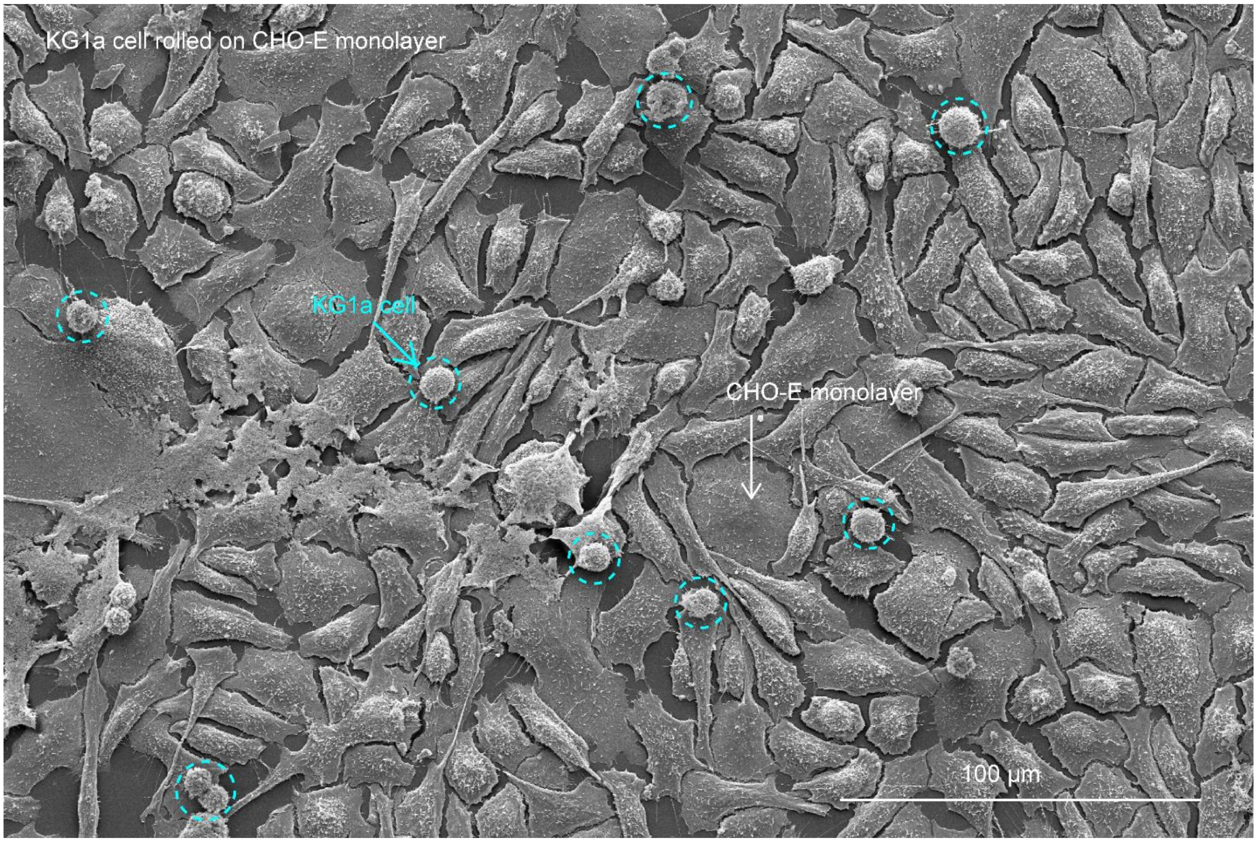

Figure S10. SEM image of KG1a cells rolled on CHO-E monolayer at the shear stress of 2 dyne cm^{-2}. KG1a cells are highlighted by circles.

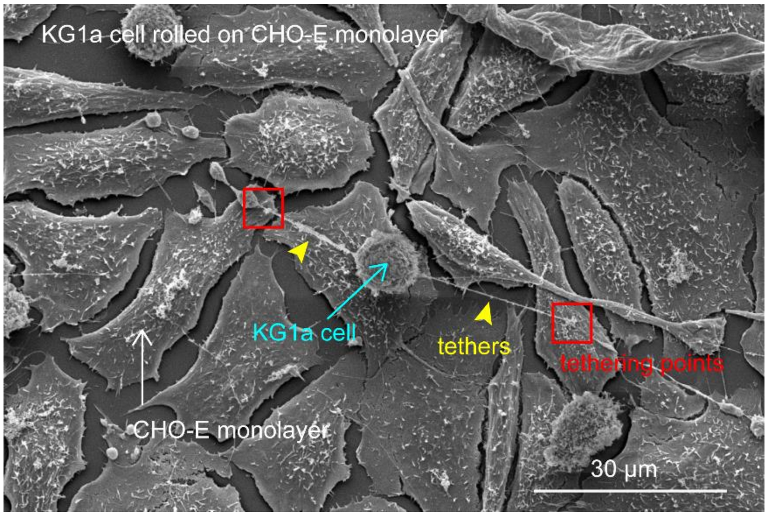

Figure S11. Enlarged view of KG1a cells rolled on CHO-E monolayer at the shear stress of 2 dyne cm^{-2}. Tethers formed during the cell rolling are highlighted by arrow heads. Tethering points are highlighted by the red squares.

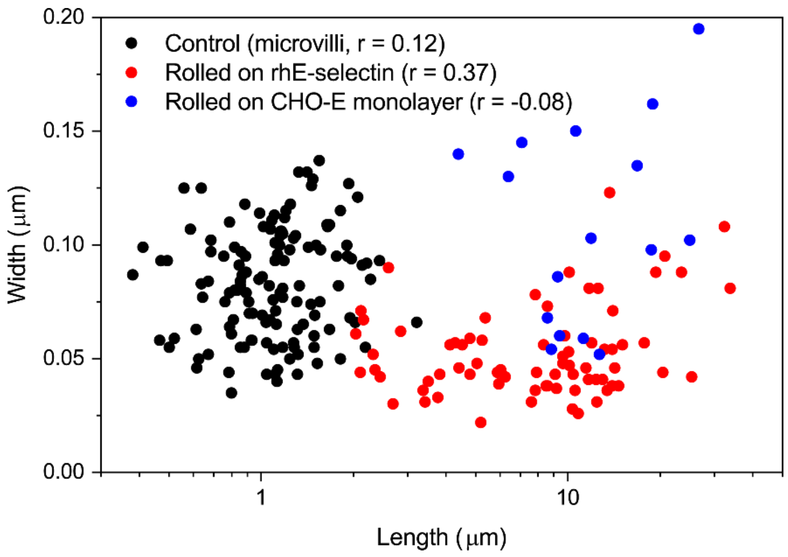

Figure S12. Correlation between the length and width of the tethers and slings formed during KG1a cells rolling on a rhE-selection coated surface (2µg ml^{-1}) (red) and on CHO-E monolayer (blue). The black dots show the correlation between the length and width of microvilli present on control KG1a cells. r denotes Pearson's correlation coefficients.

Captions for Supporting Videos

Video S1: Time-lapse bright-field microscopy images of KG1a cells rolling over CHO-E monolayer at the shear stress of 2 dyne cm^{-2}.

Video S2: Time-lapse bright-field microscopy images of KG1a cells rolling over CHO-E monolayer at the shear stress of 2 dyne cm^{-2} in the presence of 10 mM EDTA in the rolling buffer.

Video S3: Time-lapse bright-field microscopy images of KG1a cells rolling over CHO-K monolayer at the shear stress of 2 dyne cm^{-2}.
